# Development and performance of a novel vasopressor-driven mortality prediction model in septic shock

**DOI:** 10.1186/s13613-018-0459-6

**Published:** 2018-11-22

**Authors:** Saraschandra Vallabhajosyula, Jacob C. Jentzer, Aditya A. Kotecha, Dennis H. Murphree, Erin F. Barreto, Ashish K. Khanna, Vivek N. Iyer

**Affiliations:** 1 Department of Cardiovascular Medicine, Mayo Clinic, 200 First Street SW, Rochester, MN 55905 USA; 2 Division of Pulmonary and Critical Care Medicine, Department of Medicine, Mayo Clinic, Rochester, MN USA; 3 Multidisciplinary Epidemiology and Translational Research in Intensive Care (METRIC) Laboratory, Mayo Clinic, Rochester, MN USA; 4 Division of Pulmonary and Critical Care Medicine, Department of Medicine, Henry Ford Hospital/Wayne State University, Detroit, MI USA; 5 Department of Health Sciences Research, Robert D. and Patricia E. Kern Center for the Science of Health Care Delivery, Mayo Clinic, Rochester, MN USA; 6 Department of Pharmacy, Mayo Clinic, Rochester, MN USA; 7 Center for Critical Care, Anesthesiology Institute, Cleveland Clinic, Cleveland, OH USA; 8 Department of Outcomes Research, Cleveland Clinic, Cleveland, OH USA

**Keywords:** Septic shock, Vasopressors, Inotropes, Norepinephrine, MAVIC model

## Abstract

**Background:**

Vasoactive medications are essential in septic shock, but are not fully incorporated into current mortality prediction risk scores. We sought to develop a novel mortality prediction model for septic shock incorporating quantitative vasoactive medication usage.

**Methods:**

Quantitative vasopressor use was calculated in a cohort of 5352 septic shock patients and compared using norepinephrine equivalents (NEE), cumulative vasopressor index and the vasoactive inotrope score models. Having best discrimination prediction, log_10_NEE was selected for further development of a novel prediction model for 28-day and 1-year mortality via backward stepwise logistic regression. This model termed ‘MAVIC’ (Mechanical ventilation, Acute Physiology And Chronic Health Evaluation-III, Vasopressors, Inotropes, Charlson comorbidity index) was then compared to Acute Physiology And Chronic Health Evaluation-III (APACHE-III) and Sequential Organ Failure Assessment (SOFA) scores in an independent validation cohort for its accuracy in predicting 28-day and 1-year mortality.

**Measurements and main results:**

The MAVIC model was superior to the APACHE-III and SOFA scores in its ability to predict 28-day mortality (area under receiver operating characteristic curve [AUROC] 0.73 vs. 0.66 and 0.60) and 1-year mortality (AUROC 0.74 vs. 0.66 and 0.60), respectively.

**Conclusions:**

The incorporation of quantitative vasopressor usage into a novel ‘MAVIC’ model results in superior 28-day and 1-year mortality risk prediction in a large cohort of patients with septic shock.

## Introduction

Sepsis is a leading public health problem with an increasing incidence in recent epidemiological data [1]. Despite recent advances, the mortality with sepsis and septic shock remains as high as 30–50% [2, 3]. Current prognostication systems for critical illness, such as the Acute Physiology And Chronic Health Evaluation-III (APACHE-III) and Sequential Organ Failure Assessment (SOFA) scores, assess end-organ damage, laboratory and physiological derangements and chronic comorbidities to aid in accurate mortality prediction [4]. The recent Sepsis-3 definitions emphasize the incorporation of the SOFA score into defining sepsis severity, including septic shock [4]. However, since the initial development of the SOFA score, advances in cardiovascular critical care have resulted in changes to the types, doses and combinations of vasoactive medications warranting a refinement of the SOFA score [5–7]. Previous work from our institution has shown that refining the cardiovascular component of the SOFA score results in significantly improved prediction of intensive care unit (ICU), hospital and 28-day mortality in patients with critical illness [5]. More importantly, their modified cardiovascular SOFA score was as reliable as the total SOFA score for mortality prediction. This highlights the critical importance of cardiovascular integrity in overall prognostication. We have previously reported that nearly 60–70% of septic patients in the ICU have concomitant cardiac and renal end-organ damage [8, 9]. Cardiovascular dysfunction in sepsis is associated with poor short-term and long-term outcomes, and the use of high-dose vasopressors appears to further worsen prognosis in these situations [2, 3, 6, 8, 10]. Circulatory failure and shock remain the most widely noted forms of cardiovascular dysfunction in patients with sepsis. Given the importance and ubiquity of vasopressor use in septic shock, we sought to develop a novel risk prognostication system using a quantitative vasoactive medication scoring system approach. [6, 7]

Using a contemporary cohort of septic shock patients, we thus sought to develop and validate a novel risk prognostication model for 28-day mortality incorporating the best discriminative ability of three contemporary vasoactive medication scoring systems. This model was then compared to existing SOFA and APACHE-III prognostic risk scores.

## Materials and methods

This was a retrospective cohort study of adult septic shock patients admitted to all ICUs at the Mayo Clinic in Rochester, Minnesota from January 1, 2010 to December 31, 2015. The characteristics of these ICUs and patient population included in this study have been described previously [11]. Briefly, the Mayo Clinic is a 2059 bed hospital over two campuses in Rochester Minnesota and has 14 sub-specialty ICUs that are staffed continually by board-certified intensivists. This study was approved by the Mayo Clinic Institutional Review Board. The Sepsis-3 criteria was used to define septic shock as sepsis requiring vasopressor use to maintain mean arterial pressure ≥ 65 mm Hg and blood lactate ≥ 2 mmol/L despite fluid resuscitation [4]. Adult (> 18 years) patients diagnosed with septic shock admitted to the ICU for ≥ 24 h were included in this study. We excluded patients who declined Minnesota research authorization and also those with repeat ICU admissions, end-of-life treatment limiting decisions and patients without post-ICU follow-up.

### Data: definitions, sources and management

Demographics, comorbidities and clinical data were automatically abstracted from the Multidisciplinary Epidemiology and Translational Research in Intensive Care Laboratory (METRIC) DataMart as previously described [2, 3, 8, 10, 12]. This customized iterative data repository automatically abstracts vital signs, infusion rates, fluid balance, urine output data, ventilator parameters, hemodynamic variables and laboratory parameters every 15 min in real time. All patients with sepsis and septic shock have blood cultures and lactate levels checked and receive 30 ml/kg intravenous fluid and antimicrobial therapy within 3 h of sepsis onset as detected by electronic search algorithm. This is a part of an ongoing quality improvement initiative in the ICUs at Mayo Clinic [13, 14]. Acute kidney injury was electronically abstracted by a customized, validated search algorithm that screens all ICU patients [15]. Total and peak vasopressor doses for the first 24 h were abstracted from the METRIC DataMart. The SOFA and APACHE-III scores are calculated at admission and at 24 h using customized algorithms. We used the 24-h SOFA and APACHE-III scores in this study. Mortality data were abstracted from the Mayo Clinic databases, the State of Minnesota electronic death certificates and the Rochester Epidemiology Project death data system [16]. Two independent reviewers (SV and AAK) reviewed the electronically abstracted variables and, when needed, performed manual chart reviews to ensure accuracy and fidelity of data.

The primary aim was to develop a novel risk stratification model incorporating vasoactive medication scoring systems in the prediction of 28-day mortality in patients with septic shock. This novel model was then compared to currently existing standard severity of illness scoring systems, i.e., SOFA and APACHE-III scores. The secondary aim was to evaluate the ability of this model to predict 1-year mortality.

### Vasoactive medication scoring systems

**Fig. 1:**
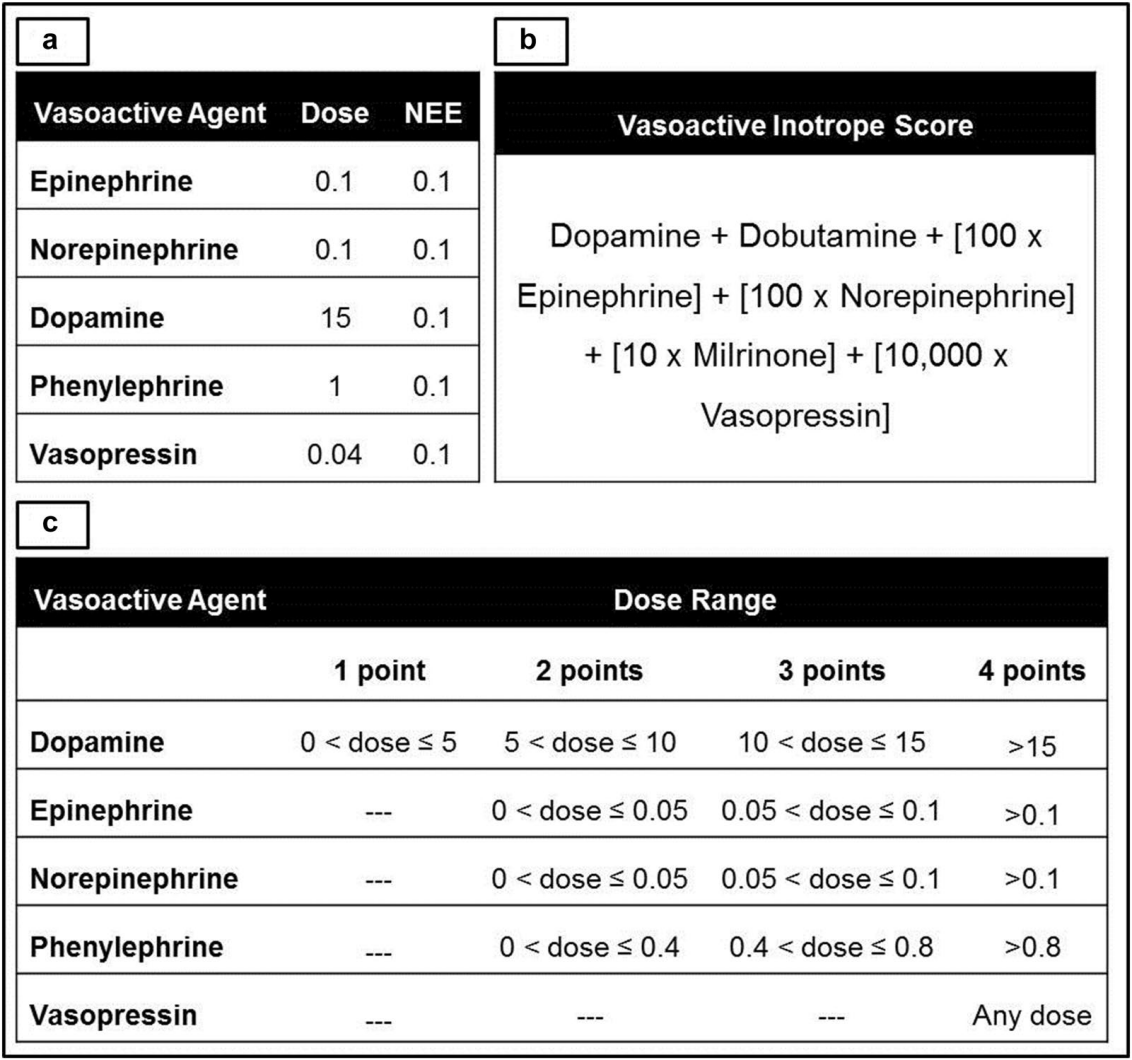
Vasoactive medication scoring systems. Conversion and scoring systems used in norepinephrine equivalents (1A), vasoactive inotrope score (1B) and cumulative vasopressor index (1C). All doses in mcg/kg/min except vasopressin, which is U/min. *NEE* norepinephrine equivalents

Total and peak simultaneous doses of vasoactive medications in the first 24-h were abstracted from the METRIC DataMart. We used the following scoring systems to quantify overall peak vasoactive medication requirements: (a) norepinephrine equivalents (NEE) [7], (b) vasoactive inotropic score (VIS) [17, 18] and (c) cumulative vasopressor index (CVI) [19]. The dose equivalency and calculations for these scoring systems are presented in Fig. 1. Peak simultaneous doses during the first 24 h of the ICU stay were used to develop these indices that were used for further analysis.

### Model development, validation and statistical analysis

Continuous and categorical data are presented as median (interquartile range [IQR]) and total (percentage), respectively. Kruskal–Wallis and Fisher’s exact tests were used to evaluate continuous and categorical outcomes. Odds ratios (OR) with 95% confidence intervals (CI) were used to report univariable and multivariable analysis results. Because of the skewed nature of NEE, VIS and CVI values, these values were converted to log_10_NEE, log_10_VIS and log_10_CVI, respectively, for continuous analyses. Area under the receiver operating characteristic curve (AUROC) analysis was utilized to evaluate discrimination of log_10_NEE, log_10_VIS and log_10_CVI for 28-day mortality on a derivation subset of the cohort. The scoring system with the highest discrimination was used further for model development in the multivariate analyses.

Using an outcome-agnostic sampling algorithm, the cohort was divided into derivation (75%) and validation (25%) subsets. As seen in Table 1, the outcomes for both 28-day and 1-year mortality are relatively balanced, thus no specific resampling approach was required. For the multivariable modeling, regression analysis with backward stepwise variable selection was conducted in order to derive a model for predicting 28-day and 1-year mortality using variables generated in the first 24 h of ICU stay. The variable selection component of this model was based on a liberal *p *< 0.20 for inclusion [20]. Following training on the derivation subset, model performance was assessed on the 25% holdout set. Two-tailed *p *< 0.05 was considered statistically significant. All statistical analyses were performed with JMP version 10.0.1 (SAS Institute, Cary, NC).

**Table 1:** Characteristics of the population at baseline and within 24 h of ICU admission

Parameter	Total cohort (*n* = 5352)	Derivation cohort (*n* = 4033)	Validation cohort (*n* = 1319)	*p*
Age (years)	65.7 (56–75.3)	65.7 (55.8–75.3)	65.9 (56.4–75.1)	0.79
Caucasian race	4879 (91.2)	3677 (91.2)	1202 (91.1)	0.49
Male sex	3297 (61.6)	2512 (62.2)	785 (59.5)	0.07
Body mass index (kg/m^2^)	28.5 (24.4–33.7)	28.5 (24.4–33.7)	28.6 (24.3–33.8)	0.88
Charlson comorbidity index	6 (4–8)	6 (4–8)	6 (3–8)	0.41
Heart failure	999 (18.7)	731 (18.1)	268 (20.3)	0.08
Diabetes mellitus, type II	1561 (29.2)	1196 (29.7)	365 (27.7)	0.17
Chronic kidney disease	1216 (22.7)	910 (22.6)	306 (23.2)	0.65
APACHE-III score (24 h)	83 (67–103)	83 (68–103)	82 (66–102)	0.39
SOFA score (24 h)	9 (7–12)	9 (7–12)	9 (8–12)	0.81
Peak lactate (mmol/L)	4.4 (2.8–7.9)	4.4 (2.8–8)	4.3 (2.8–7.8)	0.49
Invasive mechanical ventilation	4381 (81.9)	3283 (81.4)	1098 (83.2)	0.14
Acute kidney injury within 24 h	4226 (82.9)	3174 (82.5)	1052 (83.9)	0.28
Cumulative fluid balance (L) (h)				
3	6.2 (2.9–10.4)	6.2 (2.9–10.4)	6.3 (3–10.4)	0.32
6	7.1 (3.6–11.5)	7 (3.6–11.5)	7.3 (3.8–11.7)	0.27
24	9.3 (5.4–14.9)	9.6 (5.7–15.6)	9.6 (5.8–15.6)	0.23
ICU length of stay (days)	3.8 (2–7.7)	3.7 (2–7.7)	3.9 (2.1–7.7)	0.09
Hospital length of stay (days)	11.1 (6.5–21.7)	11 (6.5–21.5)	11.6 (6.6–22.2)	0.16
Peak 24-h vasopressor doses				
Norepinephrine (mcg/kg/min)	0.1 (0.16–0.25)	0.1 (0.05–0.25)	0.1 (0.05–0.25)	0.53
NEE (mcg/kg/min)	0.2 (0.1–0.32)	0.2 (0.1–0.32)	0.2 (0.1–0.32)	0.82
Vasoactive medication scores				
Log_10_NEE	− 0.7 (− 1, − 0.5)	− 0.7 (− 1, − 0.5)	− 0.7 (− 1, − 0.5)	0.82
Log_10_VIS	1.3 (1–1.5)	1.2 (1–1.5)	1.2 (1–1.5)	0.95
Log_10_CVI	0.7 (0.6–0.9)	0.7 (0.6–0.9)	0.8 (0.6–0.9)	0.25
≥ 2 vasoactive medications	3245 (60.6)	2412 (60.5)	833 (60.9)	0.80
28-day mortality	2416 (45.1)	1842 (45.7)	574 (43.5)	0.18
One-year mortality	2660 (49.7)	2026 (50.2)	634 (48.1)	0.17

Count (percentage) or median (interquartile range)

*APACHE* Acute Physiology And Chronic Health Evaluation, *CVI* cumulative vasopressor index, *ICU* intensive care unit, *NEE* norepinephrine equivalents, *SOFA* Sequential Organ Failure Assessment, *VIS* vasoactive inotropic score

## Results

**Fig. 2:**
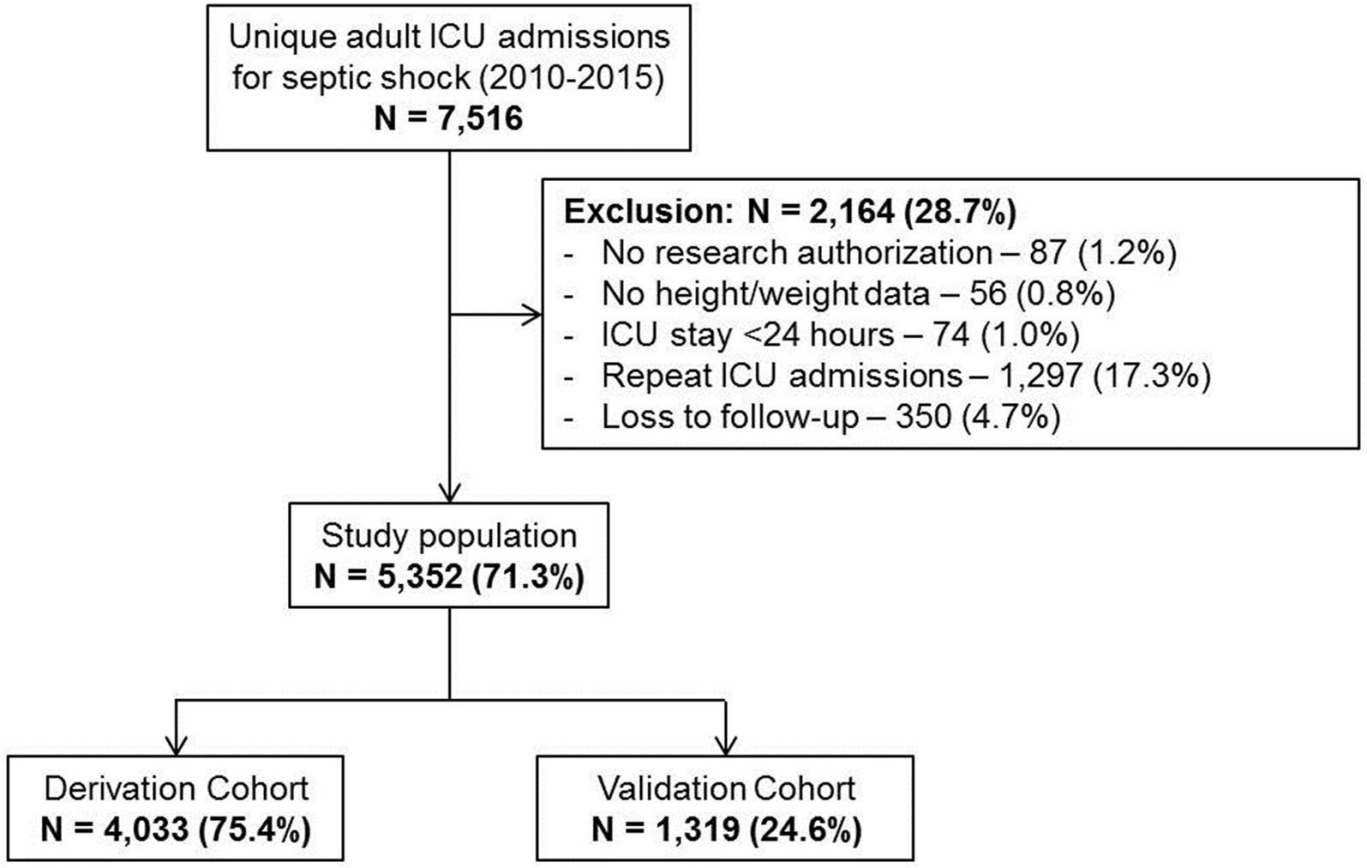
Study population. *ICU* intensive care unit

**Fig. 3:**
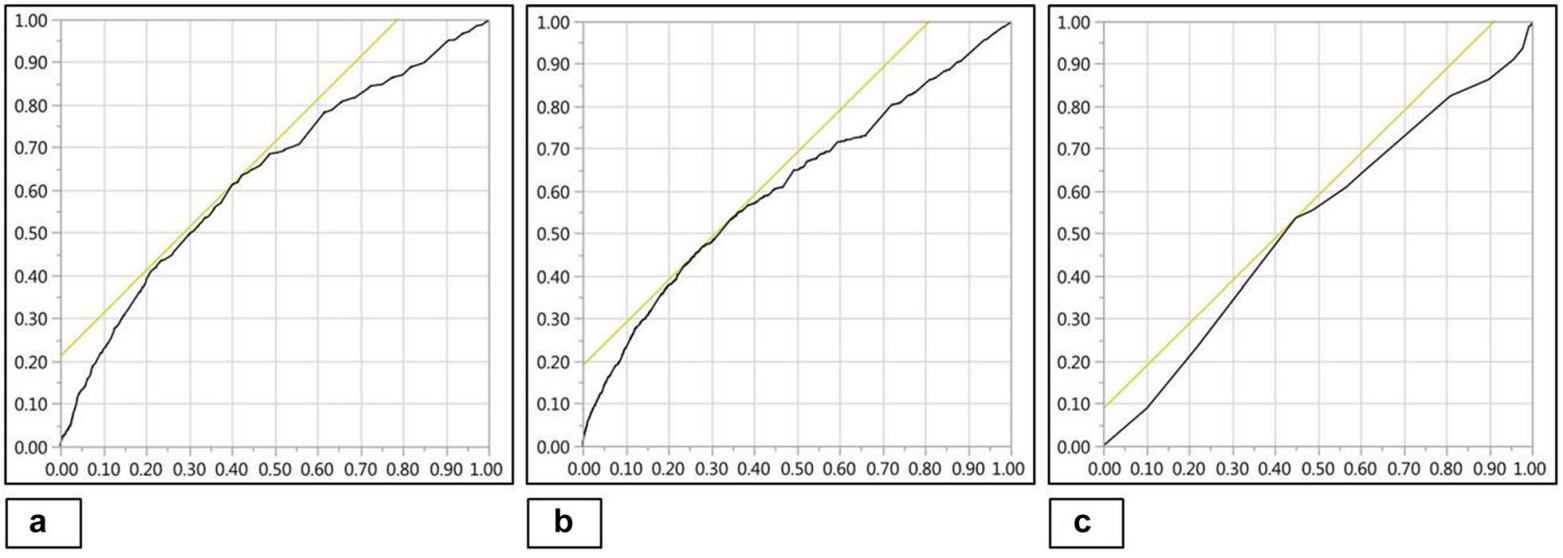
Discrimination of vasoactive scoring systems for 28-day mortality. AUROC curve for log_10_NEE = 0.63 (**a**); log_10_VIS = 0.61 (**b**); and log_10_CVI = 0.52 (**c**). *AUROC* area under receiver operating characteristic, *CVI* cumulative vasopressor index, *NEE* norepinephrine equivalents, *VIS* vasoactive inotrope score

During the period from January 1, 2010 through December 31, 2010, there were a total of 7516 ICU admissions for septic shock, of which 5352 (71.3%) patients were included (Fig. 2). The derivation and validation cohorts comprised 4033 (75.4%) and 1319 (24.6%) patients, respectively. Baseline characteristics for the total population and the individual cohorts are detailed in Table 1. The median vasopressor doses and vasoactive medication scores were comparable between the two cohorts (Tables 1, 2). In the total population, 28-day and 1-year mortalities were 45.1% and 49.7%, respectively, there were no significant differences in mortality between the derivation and validation cohorts (Table 1). All three vasoactive medication scoring systems were associated with unadjusted 28-day mortality in the derivation cohort—log_10_NEE (OR 2.3 [95% CI 2.1–2.6]; *p *< 0.001), log_10_VIS (OR 2.9 [95% CI 2.5–3.3]; *p *< 0.001) and log_10_CVI (OR 1.2 [95% CI 1.1–1.2]; *p *< 0.001). Log_10_NEE and log_10_VIS demonstrated fair discrimination (0.63, 0.61) prediction and log_10_CVI demonstrated poor discrimination (0.52) for 28-day mortality in derivation cohort (Fig. 3). Log_10_NEE was used for further analysis in the multivariate model and for the development of a prediction model.

**Table 2:** Peak vasoactive medication dosing in first 24 h

Vasoactive medication	Total cohort (*n* = 5352)	Derivation cohort (*n* = 4033)	Validation cohort (*n* = 1319)	*p*
Vasopressin				
Total patients	2200 (41.1)	1644 (40.8)	556 (42.2)	0.38
Peak dose (U/min)	0.04 (0.04–0.04)	0.04 (0.04–0.04)	0.04 (0.04–0.04)	0.98
Epinephrine				
Total patients	1633 (30.5)	1207 (29.9)	426 (32.3)	0.11
Peak dose (mcg/kg/min)	0.05 (0.04–0.1)	0.05 (0.04–0.1)	0.05 (0.04–0.1)	0.47
Phenylephrine				
Total patients	355 (6.6)	280 (6.9)	75 (5.7)	0.13
Peak dose (mcg/kg/min)	0.6 (0.1–1.3)	0.6 (0.3–1.2)	0.8 (0.4–1.4)	0.10
Dobutamine				
Total patients	271 (5.1)	201 (5.0)	70 (5.3)	0.66
Peak dose (mcg/kg/min)	5 (5–10)	5 (5–10)	5 (5–7.5)	0.38
Dopamine				
Total patients	208 (3.9)	150 (3.7)	58 (4.4)	0.29
Peak dose (mcg/kg/min)	5 (3–10)	5 (3–10)	5 (3–13)	0.29
Milrinone				
Total patients	700 (13.1)	522 (12.9)	178 (13.5)	0.61
Peak dose (mcg/kg/min)	0.25 (0.25–0.37)	0.25 (0.25–0.38)	0.25 (0.2–0.3)	0.12

Total (percentage) or median (interquartile range)

### Model development, validation and comparison

**Fig. 4:**
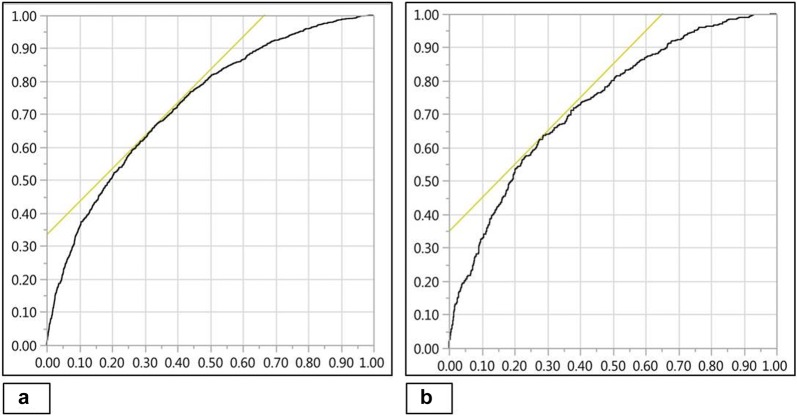
Discriminatory ability of MAVIC model for 28-day mortality. AUROC curve for derivation (**a**) and validation cohorts (**b**) = 0.73. *AUROC* area under receiver operating characteristic, *MAVIC* Mechanical ventilation, APACHE-III, Vasopressors, Inotropes and Charlson comorbidity index

Using univariable analysis, significant predictors of 28-day mortality in the derivation cohort are identified as noted in Table 3. After adjusting for age, sex, severity of illness, comorbidity and use of mechanical ventilation, log_10_NEE was an independent predictor of 28-day mortality (OR 1.6 [95% CI 1.4–1.9]; *p *< 0.001). The multivariate model was reduced using a stepwise backward elimination process based on a liberal *p *< 0.20. The final model incorporated Mechanical ventilation, APACHE-III, Vasopressors and Inotropes (log_10_NEE) and Charlson comorbidity index (MAVIC) as significant predictors of 28-day mortality in patients with septic shock. This model demonstrated good discrimination of 28-day mortality (AUROC 0.73) in the validation cohort (Fig. 4). In comparison with the MAVIC model, standardized severity of illness scoring systems such as the APACHE-III (AUROC 0.66) and SOFA score (AUROC 0.59) showed lower discrimination for 28-day mortality. Formal comparisons of all model AUROCs via DeLong test found all *p *< 0.001, indicating statistically significant differences.

**Table 3:** 28-day mortality predictors in the derivation cohort

Parameter	Univariable analysis		Multivariable analysis		MAVIC model	
	OR (95% CI)^a^	*p*	OR (95% CI)^a^	*p*	OR (95% CI)^a^	*p*
Age	1.1 (1.1–1.1)	< 0.001	1.0 (0.9–1.0)	0.88	–	–
Male sex	0.8 (0.7–0.9)	0.001	0.9 (0.8–0.9)	0.03	–	–
BMI (kg/m^2^)	0.9 (0.9–0.9)	0.02	0.9 (0.9–0.9)	< 0.001	–	–
CCI	1.2 (1.2–1.2)	< 0.001	1.2 (1.1–1.2)	< 0.001	1.1 (1.1–1.2)	< 0.001
APACHE-III score	1.1 (1.1–1.1)	< 0.001	1.1 (1.1–1.1)	< 0.001	1.1 (1.1–1.1)	< 0.001
Peak lactate (mmol/L)	1.1 (1.1–1.1)	< 0.001	1.1 (1.1–1.1)	< 0.001	–	–
Acute kidney injury	2.1 (1.7–2.5)	< 0.001	1.9 (1.6–2.3)	< 0.001	–	–
IMV	1.6 (1.4–1.9)	< 0.001	1.8 (1.5–2.2)	< 0.001	1.8 (1.5–2.2)	< 0.001
24-h cumulative fluids	1.0 (0.9–1.0)	0.77	–	–	–	–
Log_10_24-h peak NEE	2.3 (2.1–2.6)	< 0.001	1.6 (1.4–1.9)	< 0.001	2.0 (1.7–2.3)	< 0.001

*APACHE-III* Acute Physiology And Chronic Health Evaluation-III, *BMI* body mass index, *CCI* Charlson comorbidity index, *CI* confidence interval, *IMV* invasive mechanical ventilation, *NEE* norepinephrine equivalents, *OR* odds ratio

^a^Unit odds ratios are represented for continuous variables

The MAVIC model, likewise, demonstrated superiority in discrimination for 1-year mortality (AUROC 0.73) as compared to APACHE-III (AUROC 0.66) and SOFA scores (AUROC 0.60) in the validation cohort. As with 28-day mortality, comparison via DeLong test found all differences to be statistically significant.

## Discussion

To the best of our knowledge, this is the first large observational study to use vasoactive scoring systems for prediction of mortality in adult patients with septic shock. Norepinephrine equivalents had superior discrimination for 28-day mortality compared to either the CVI or VIS in septic shock patients. Using data available within the first 24 h, NEE and three other independent predictors of 28-day mortality were combined to develop a parsimonious 4-component prognostic model (MAVIC). This MAVIC model was subsequently validated in a randomly generated validation cohort from the same population. The MAVIC model demonstrated superior discriminatory ability compared to the APACHE-III and SOFA scores for 28-day and 1-year mortality in septic shock.

Historically, in adult patients with sepsis and cardiogenic shock, vasoactive medication scoring systems have predominantly been used to classify the extent of hemodynamic support [21–23]. Vasoactive medications are used in an empiric fashion and often there are limited bedside data on cumulative vasopressor requirements [24]. Vasoactive medications are a severity of illness indicator; however, they are incompletely accounted for in scoring systems. More recent data have shown vasoactive medication requirements to be an independent predictor of adverse outcomes [11, 25–27]. The need for high-dose vasopressors reflects a potentially fatal underlying condition with a high risk of complications, but the direct association remains to be proven. Though the VIS has been used to prognosticate in a smaller cohort of 138 pediatric patients with sepsis [28], this is the first large study using existing vasoactive scoring systems in the prognostication of adult septic shock patients. Prior studies have used either norepinephrine or cumulative vasopressor requirements to demonstrate higher mortality in patients with septic shock [27, 29]. In about 20–40% of septic patients, persistent vasoplegia results in refractory shock necessitating high-dose vasoactive medications, with a very high attributable mortality [29]. Refractory vasoplegia in sepsis is multi-factorial and usually fatal, leading investigators to evaluate alternate mechanisms of restoring vascular tone in these patients [6, 30]. High doses of vasopressors are associated with significant adverse events such as arrhythmias, myocardial infarctions, digital injury/ischemia and acute kidney injury [26, 27].

Existing prognostic scores (APACHE and SOFA) have several limitations [31]. The APACHE-III does not incorporate either vasopressor use or myocardial dysfunction in sepsis, both of which have mortality implications [2, 3, 8, 11]. The SOFA score also has some similar limitations as shown in the study by Yadav et al. [5]. They modified the cardiovascular component of the SOFA score and incorporated more detailed vasoactive medication dosage as well as organ failure assessment using serum lactate and the shock index. Their modified cardiovascular SOFA showed superior discriminatory power for ICU, in-hospital as well as 28-day mortality [5]. This study was limited in its ability to prognosticate in septic shock patients since it included all ICU patients. We performed an exploratory analysis using the modified cardiovascular SOFA in this population and it showed a poor discrimination for 28-day mortality (AUROC 0.51). This can partly be explained by the high modified cardiovascular SOFA score of 3 and 4 in this population reflective of their cardiovascular morbidity. Furthermore, the NEE model uses a more granular scoring system compared to the modified cardiovascular SOFA that might explain these differences. In another study, Johnson et al. [32] used a simplified version of the APACHE-IV score and developed a similar parsimonious scoring system in unselected critically ill patients without compromising the accuracy; however, it lacked adequate vasopressor data.

It is noteworthy that our model appears to further enhance risk assessment in patients with septic shock, who by definition comprise a high-risk cohort with a cardiovascular SOFA score of 3–4. Thus, our model appears to capture additional prognostic information than the individual SOFA component scores. Furthermore, with the advent of real-time sophisticated critical-illness computational algorithms, our model appears capable of incorporating real-time vasopressor use to provide clinicians with reliable and up-to-date prognostic information [12, 33].

The vasoactive scoring systems used in this study have been previously been used in the evaluation of septic shock patients; however, they have not been compared head-to-head [7, 17, 19]. In this study, we demonstrate NEE to have superior predictive performance for 28-day mortality relative to the VIS and CVI. This can be explained by multiple hypotheses. First, given the ubiquitous use of norepinephrine as the first-line vasopressor in septic shock it is likely that norepinephrine usage dominates the various scoring systems [34]. As noted in Table 1, individual peak norepinephrine doses formed nearly 40% of the peak total dose of NEE lending credence to this hypothesis. Second, the VIS scoring system was used and validated primarily to define inotrope requirements in pediatric and cardiac surgery populations, both of which significantly differ from the current population [17, 28]. Finally, the dose equivalency in the literature differs on the optimal conversion factors for vasoactive medications resulting in varied calibration with outcomes. The NEE conversion system used in this study is consistent with recent high-impact trials including the angiotensin II for the treatment of vasodilatory shock and vasopressin versus norepinephrine infusion in patients with septic shock trials [7, 35]. However, the NEE used by Brown et al. [29] to report high-dose vasopressors differs from our study.

This study has certain limitations. The use of a retrospective design is subject to selection and information bias that could have confounded the results. These patients were identified using the Sepsis-3 criteria retroactively during the study duration when the 2001 consensus criteria were in use; and this may have impacted case selection. All treatment decisions, including choice of vasoactive medication and arterial pressure goal, were at the discretion of the treating physician and no universal protocol for hemodynamic management of septic patients was in place during the study period. Despite protocoled hemodynamic management, a significant portion of the patients had a positive fluid balance at the end of day 1 suggestive of higher acuity of illness in this population. Alternate endpoints to critical illness such as functional status, disposition and return to work were not assessed in this study that could provide a greater emphasis on patient-centered outcomes and quality of life. We focused on early risk prognostication and thus could have overlooked late cardiovascular deterioration in these patients that could have contributed to 28-day mortality. Finally, the single-institution nature of this study limits external generalizability in the absence of validation studies at other centers. Strengths of the study include the large patient population studied and the use of a well-validated electronic database with 15-min data capture capabilities.

## Conclusions

We report the development and validation of a novel MAVIC mortality prediction model for septic shock patients admitted to the ICU. The MAVIC model incorporates cumulative vasoactive medication usage within the first 24 h and outperforms both APACHE-III and SOFA scores for 28-day mortality prediction in septic shock patients. Vasopressor burden during early critical illness appears to be a marker for unfavorable outcomes. Newer scoring systems for critically ill patients with septic shock are needed that emphasize cardiovascular morbidity and vasoactive medication use to aid in early and reliable prognostication. Further validation of this model externally and in non-septic shock populations is desirable prior to acceptance in clinical practice.

## Abbreviations

APACHE-III: Acute Physiology And Chronic Health Evaluation-III
AUROC: area under receiver operating characteristic curve
CI: confidence interval
CVI: cumulative vasopressor index
ICU: intensive care unit
MAVIC: Mechanical ventilation, APACHE-III, Vasopressors, Inotropes, Charlson comorbidity index
METRIC: Multidisciplinary Epidemiology and Translational Research in Intensive Care Laboratory
NEE: norepinephrine equivalents
OR: odds ratio
SOFA: Sequential Organ Failure Assessment
VIS: vasoactive inotrope score
